# Predicting apheresis yield and factors affecting peripheral blood stem cell harvesting using a machine learning model

**DOI:** 10.1177/03000605241305360

**Published:** 2024-12-24

**Authors:** Jing Qi, Yinchu Chen, Xiaoke Jin, Ran Wang, Nana Wang, Jiawei Yan, Chen Huang, Jun Huang, Yuanfeng Wei, Faqin Xie, Zhengzhi Yu, Dongping Huang

**Affiliations:** 1 Department of Hematology, 569222 The First Affiliated Hospital of Wannan Medical College, Wuhu, Anhui, China

**Keywords:** Peripheral blood stem cell, mobilization, apheresis, machine learning model, prediction, logistic regression

## Abstract

**Objective:**

Mobilization and collection of peripheral blood stem cells (PBSCs) are time-intensive and costly. Excessive apheresis sessions can cause physical discomfort for donors and increase the costs associated with collection. Therefore, it is essential to identify key predictive factors for successful harvests to minimize the need for multiple apheresis procedures.

**Methods:**

We retrospectively analyzed 88 PBSC donations at our hospital. Mobilization involved disease-specific chemotherapy plus human recombinant granulocyte-colony-stimulating factor (G-CSF; lenograstim) or G-CSF alone for 5 days, followed by apheresis on day 5. The baseline characteristics of donors, pre-apheresis complete blood counts, and CD34+ cells were evaluated. Univariate logistic regression, the eXtreme Gradient Boosting algorithm, and multivariate logistic regression were applied to select significant predictive variables. The multivariate logistic regression results were integrated into various machine learning models to assess predictive accuracy.

**Results:**

The percentage of pre-collection monocytes (Mono%), age, and CD34+ cell percentage (CD34+ cell%) were identified as significant independent factors that could accurately predict the success of an initial PBSC harvest.

**Conclusions:**

We used machine learning methods to identify and validate Mono%, age, and CD34+ cell% as significant factors predictive of successful PBSC harvest on the first attempt, offering important insight to guide the clinical harvesting of PBSCs.

## Introduction

Hematopoietic stem cell transplantation (HSCT) is an important method for treating various malignant hematological diseases, such as acute leukemia, lymphoma, and multiple myeloma.^1,2^ The current sources of hematopoietic stem cells mainly include peripheral blood hematopoietic stem cells (PBSCs), umbilical cord blood, and bone marrow hematopoietic stem cells (BMSCs).^3,4^ Mobilization of stem cells from bone marrow to the peripheral blood using granulocyte-colony-stimulating factor (G-CSF) can lead to a higher yield of stem cells in a short period.^5^ Additionally, collection is less invasive than the surgical procedure required to harvest BMSCs, with reduced recovery time, which makes PBSC collection more donor-friendly. Thus, PBSCs have been a preferred choice for stem cell mobilization in many therapeutic contexts. Even so, in some patients and healthy donors, a sufficient amount of CD34+ PBSCs cannot be obtained in one apheresis.^6^ Mobilization and apheresis remains a time-consuming and expensive process. Excessive apheresis can cause physical discomfort to donors and can also increase the collection cost. Therefore, factors to effectively predict the success of harvesting must be identified to reduce the number of PBSC apheresis sessions.

Previous studies have revealed factors affecting the success of apheresis, including the general condition of donors, the method of mobilization, and the process of collection.^7–9^ Recently, Kayser et al.^7^ showed that older age, female sex, white blood cells (WBC) <30 × 10^9^/L after 5 days of G-CSF mobilization, and a donor/recipient weight ratio <1 are valuable markers of suboptimal mobilization in healthy allogeneic donors. Additionally, a simple model has been developed using age, sex, lactate dehydrogenase (LDH) on day 4, and red blood cell count at baseline to predict the optimal date to avoid multiple harvests in healthy donors.^8^ In another study, a simple predictive model was developed to estimate the BV (blood volume) to process based on the level of CD34+ cells before collection.^9^ These studies hold important implications for avoiding multiple collections and reducing collection costs.

In our present work, we conducted a retrospective, single-center analysis of PBSC collection events using machine learning, which incorporated the baseline condition of donors (autologous transplant patients and allogeneic healthy donors) and complete blood parameters as well as the CD34+ cell% before collection. Our goal was to identify critical factors that are predictive of successful collection on the first attempt, irrespective of whether the donors were autologous transplant patients or healthy donors. The aim was to enhance effectiveness of the PBSC collection process and reduce the need for subsequent harvests.

## Methods

### Donors

We retrospectively analyzed allogeneic and autologous PBSC donations performed at the Hematology Department of the First Affiliated Hospital of Wannan Medical College from 2012 to 2021. This study included 42 allogeneic healthy donors and 46 autologous transplant patients with hematological malignancies (21 patients with lymphoma, 21 patients with multiple myeloma, and four patients with acute leukemia). All donor details were de-identified to ensure privacy and confidentiality. Owing to the retrospective nature of this study, ethics approval and the requirement for patient informed consent were waived by the First Affiliated Hospital of Wannan Medical College in accordance with national legislation and institutional requirements. This study was conducted in accordance with the Helsinki Declaration of 1975 as revised in 2013^10^ and with the TRIPOD guideline.^11^

### Mobilization and apheresis

A complete medical history and examination were performed to exclude organ failure and infectious diseases before donation. A physical examination, complete blood count, serum chemistry, coagulation tests, infectious tests, electrocardiogram, and chest computed tomography or X-ray before donation were carried out.

In autologous transplant patients, disease-specific chemotherapy was administered, followed by human recombinant G-CSF (lenograstim; Granocyte™, Chugai Pharmaceutical Co., Ltd. Chuo-ku, Tokyo, Japan) at a dose of 10 µg/kg of the donor’s body weight (b.w.) subcutaneously once daily for 5 consecutive days. Healthy donors received the same G-CSF alone, at 10 µg/kg b.w. subcutaneously. Complete blood count and CD34+ cells were evaluated before apheresis on day 5. In our study, apheresis was not triggered based on specific variable thresholds. Apheresis was initiated 2 hours after the administration of G-CSF on day 5 through either the cubital vein or a central venous catheter (CVC) using the Fresenius ComTec cell separator (Fresenius Kabi, Nanchang, China). The autoMNC program was chosen, and the P1YA kit was used (Shanghai Jumu Medical Equipment Co., Ltd., Shanghai, China). The processed blood volume (PBV) was calculated based on a previous study.^9^ Acid citrate dextrose solution A was used, and oral calcium gluconate hydrate was administered during the procedure. Complete blood count and electrolytes were examined after apheresis.

### CD34+ cell analysis

The cell counts were analyzed using Cell Dyn 3700 (Abbott Diagnostics, Abbott Park, IL, USA). CD34+ cells in the apheresis products were considered related to the total mononuclear cells and were identified by staining with anti-human monoclonal CD34-APC antibodies and CD45-PerCP antibodies (BD Biosciences, Shanghai, China) in flow cytometry (BD FACSCanto™, Shanghai, China). A failed first apheresis was defined as not meeting the minimum threshold of CD34+ cells in the harvested product, 2 × 10^6^/kg of the recipient’s b.w., suggesting that a second apheresis should be performed.^12^

### Statistical analysis

All statistical analyses were performed using R software version 4.3.2 (www.r-project.org). Clinical variables were incorporated into a univariate logistic regression model to identify potential factors influencing the success of a single collection in patients and healthy donors. Subsequently, significant univariate results were subjected to feature importance ranking using the eXtreme Gradient Boosting (XGBoost) algorithm.^13^ The univariate results were then incorporated into a multivariate logistic regression model to further identify independent factors. The results of multivariate logistic regression were integrated into various machine learning models. Subsequently, 60% of cases were randomly selected as a validation cohort, and the receiver operating characteristic (ROC) curve was used to evaluate the predictive value of these variables in determining the success of the initial PBSC harvest.

## Results

### Donor baseline characteristics

In this study, we analyzed the baseline characteristics of 88 donors enrolled for PBSC harvest. The average age of donors in the failure group was 46.36 years, slightly higher than the 39.44 years in the success group, although this difference was not statistically significant. The sex distribution showed more men (63.6%) in the failure group than in the success group (77.9%). Significant differences were observed in body surface area (BSA), with the failure group having a lower average BSA (1.66 m^2^) compared with the success group (1.82 m^2^, p = 0.032). Similarly, body mass index (BMI) classifications also differed significantly between the groups (p = 0.044), with a higher proportion of donors with high BMI in the success group. Blood group distribution and disease status differed significantly; notably, all donors in the failure group had an underlying disease (100%) and received disease-specific chemotherapy (100%); most participants in the success group were healthy (54.5%, p = 0.002). Venous access significantly influenced the collection outcomes, with peripheral access associated with higher success rates than CVC (p = 0.005). Laboratory results showed significantly lower values in the failure group for WBC, hemoglobin (HGB), platelet count (PLT), and hematocrit (HCT), with all p-values <0.001. Additionally, pre-collection monocytes (Mono%) and the monocyte count were higher in the successful group, with a significant difference also noted in LDH levels (p = 0.001) (Table 1). The PBV during the collection procedure was 12,614 ± 2548 mL, and the collection time was 266 ± 58 minutes. Overall, these findings suggest that baseline physical health, particularly factors like BSA and BMI, alongside laboratory indices, are predictive of the success of stem cell harvest in donors.

**Table 1.** Baseline characteristics of enrolled donors who underwent peripheral blood stem cell harvest.

Characteristics	Overall (N = 88)		p
	Failure (%)n = 11	Success (%)n = 77	
Age (years)	46.36 ± 13.30	39.44 ± 13.21	0.108
Sex			0.508
Female	4 (36.4)	17 (22.1)	
Male	7 (63.6)	60 (77.9)	
BSA	1.66 ± 0.16	1.82 ± 0.23	0.032
BMI (kg/m^2^)			0.044
Low	0 (0.0)	3 (3.9)	
Medium	8 (72.7)	26 (33.8)	
High	3 (27.3)	48 (62.3)	
Blood			0.584
A	4 (36.4)	24 (31.2)	
AB	0 (0.0)	11 (14.3)	
B	3 (27.3)	15 (19.5)	
O	4 (36.4)	27 (35.1)	
Disease status			0.002
Disease	11 (100.0)	35 (45.5)	
Healthy	0 (0.0)	42 (54.5)	
Mobilization			0.002
G-CSF + chemo	11 (100.0)	35 (45.5)	
G-CSF	0 (0.0)	42 (54.5)	
Venous access			0.005
Peripheral	6 (54.5)	70 (90.9)	
CVC	5 (45.5)	7 (9.1)	
WBC (×10^9^/L)	3.71 ± 2.26	30.91 ± 23.63	<0.001
HGB (g/dL)	93.00 ± 11.42	126.75 ± 25.89	<0.001
PLT (×10^9^/L)	53.45 ± 14.16	137.82 ± 61.86	<0.001
Reticulocytes (×10^6^/L)	0.01 ± 0.01	0.01 ± 0.02	0.708
HCT (%)	0.28 ± 0.04	0.38 ± 0.09	0.001
Monocytes (%)	5.84 ± 4.99	12.65 ± 8.38	<0.001
Monocytes (×10^9^/L)	0.19 ± 0.16	3.7 ± 5.32	<0.001
Lymphocytes (%)	26.44 ± 29.68	12.30 ± 13.54	0.008
LDH (U/L)	149.97 ± 71.92	359.23 ± 195.97	0.001
CD34+ (%)	0.27 ± 0.28	0.80 ± 0.82	0.037

BSA, body surface area; BMI, body mass index; GSF, granulocyte-colony-stimulating factor; HCT, hematocrit; LDH, lactate dehydrogenase; HGB, hemoglobin; WBC, white blood cells; chemo; disease-specific chemotherapy; CVC, central venous catheter; PLT, platelets.

### Univariate logistic regression to identify factors influencing success of initial PBSC collection

Seventeen variables were included in this study, such as age, sex, weight, height, BMI, BSA, blood type, and pre-collection parameters (WBC, HGB, PLT, reticulocytes, HCT, Mono%, percentage lymphocytes [Lym%], LDH, and CD34+ cell%) in peripheral blood. The results of univariate logistic regression indicated that nine variables—age, BMI, WBC, HGB, PLT, Mono%, Lym%, LDH, and CD34+ cell%—were significant factors affecting the success of PBSC collection (Table 2).

**Table 2.** Univariate logistic regression analysis of enrolled variables to predict successful PBSC collection.

Factors	β	SE	Z-value	OR	95% CI	p
Age	−0.076	0.033	−2.284	0.927	0.868–0.989	0.022
BMI	0.318	0.136	2.338	1.374	1.052–1.794	0.019
WBC (10^9^/L)	0.459	0.232	1.983	1.583	1.005–2.492	0.047
HGB (g/L)	0.073	0.021	3.518	1.076	1.033–1.121	0.000434
PLT (10^9^/L)	0.032	0.010	3.169	1.033	1.012–1.053	0.00153
Monocytes (%)	0.876	0.299	2.934	2.402	1.337–4.312	0.00334
Lymphocytes (%)	1.081	0.304	3.556	2.949	1.624–5.352	0.000377
LDH (IU/mL)	0.027	0.008	3.505	1.027	1.011–1.042	0.000457
CD34^+^ (%)	1.338	0.672	1.993	3.813	1.022–14.217	0.046

PBSC, peripheral blood stem cells; SE, standard error; OR, odds ratio; CI, confidence interval; BMI, body mass index; LDH, lactate dehydrogenase; WBC, white blood cells; PLT, platelets.

### Independent factors influencing success of the initial PBSC collection using the XGBoost algorithm and multivariate logistic regression

**Figure 1.**
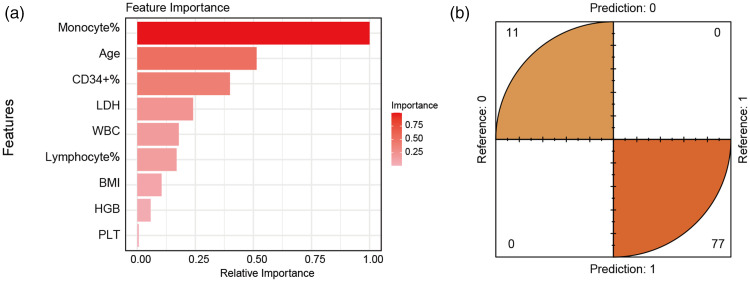
Identification of important factors influencing success of initial PBSC collection using the XGBoost Algorithm. (a) Mono%, age, and CD34^+^ cell% emerged as the three leading variables in terms of importance and (b) results of the confusion matrix suggested that the three leading variables selected using XGBoost can effectively differentiate between positive and negative outcomes of the first PBSC collection. XGBoost, eXtreme Gradient Boosting; PBSC, peripheral blood stem cells; Mono%, percentage of pre-collection monocytes; LDH, lactate dehydrogenase; WBC, white blood cells; BMI, body mass index; HGB, hemoglobin; PLT, platelets.

To further identify independent factors influencing the success of PBSC collection, application of the XGBoost algorithm yielded important findings. Mono%, age, and CD34+ cell% emerged as the three most important features (Figure 1(a)). These three variables (Mono%, age, CD34+ cell%) contributed significantly to the model’s ability to predict successful PBSC collection (Figure 1(b)). Subsequent analysis using multivariate logistic regression confirmed that Mono%, age, and CD34+ cell% remained independent factors significantly influencing the success of PBSC collection (Table 3). These findings underscore the crucial role of specific hematological parameters and donor age in the PBSC collection process.

**Table 3.** Multivariate logistic regression analysis of significant independent variables to predict successful PBSC collection.

Factors	β	SE	Z-value	OR	95% CI	p
Age	−0.095	0.044	−2.177	0.909	0.834–0.990	0.029
Monocytes (%)	0.993	0.354	2.805	2.699	1.348–5.401	0.005
CD34^+^ (%)	1.881	0.807	2.329	6.557	1.347–31.912	0.019

PBSC, peripheral blood stem cells; SE, standard error; OR, odds ratio; CI, confidence interval.

### Mono%, age, and CD34+ cell% could accurately forecast the success of an initial PBSC collection

**Figure 2.**
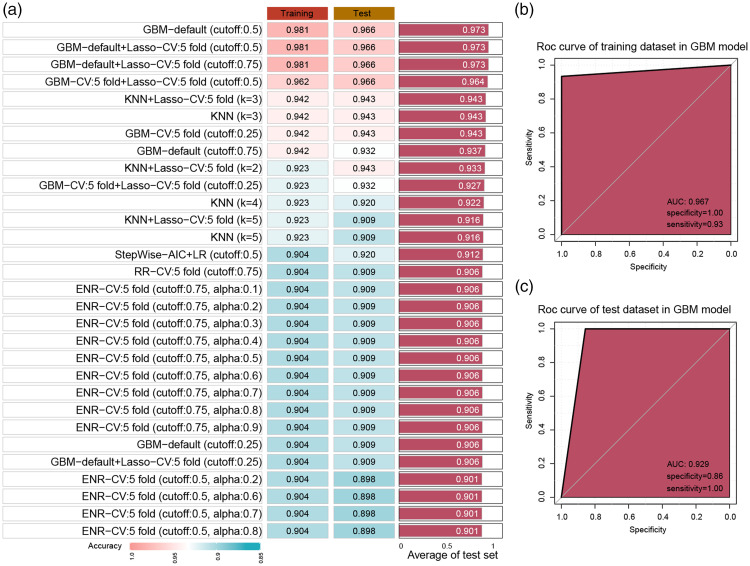
Machine learning models to validate the accuracy of independent factors in predicting successful PBSC collection. (a) Accuracy values obtained from the 30 leading cases predicted using Mono%, age, and CD34^+^ cell% with multiple algorithm combinations are presented in the bar plot. (b) ROC curve showing the predictive ability of the GBM model in the training set and (c) ROC curve showing the predictive ability of the GBM model in the test set. PBSC, peripheral blood stem cells; Mono%, percentage of pre-collection monocytes; GBM, ROC, receiver operating characteristic; AUC, area under the ROC curve; KNN, K-nearest neighbor; LR, logistic regression; RR, ridge regression; ENR, elastic net regression.

To validate whether the factors Mono%, age, and CD34+ cell% can accurately predict the success rate of donor PBSC collection, these three variables were input into various machine learning models. Sixty percent of cases were randomly selected as a validation set. Figure 2(a) displays the accuracy values obtained from the 30 leading cases using multiple algorithm combinations. Inputting these three variables into a gradient boosting machine (GBM) model, we could accurately predict the success rate of a single PBSC collection, achieving an accuracy of 0.981 with an area under the ROC curve (AUC) of 0.967 in the training set (Figure 2(a), 2(c)) and an accuracy of 0.966 and AUC of 0.929 in the test set (Figure 2(a), 2(c)).

## Discussion

Over the past few decades, PBSCs have been widely used in the field of HSCT.^14^ The mobilization and collection process begins with the donor receiving a course of growth factors, typically G-CSF, to encourage migration of stem cells from the bone marrow into the peripheral blood.^15^ The suggested dosage of G-CSF is 10 μg/kg/day subcutaneously for 4 to 5 consecutive days,^12,16^ with the target yield of CD34+ cells being 4 to 5 × 10^6^/kg of the recipient’s b.w.^12^ The acceptable minimum value is 2 × 10^6^/kg b.w. in both autologous and allogeneic settings.^17^ Leukapheresis typically takes place on day 5 after the administration of G-CSF in most centers, which aligns with our practice.^4^ However, some scholars have suggested that sufficient CD34+ cells might already be obtainable by the day 4 of mobilization,^8,18^ potentially leading to sustained hematopoiesis for the recipient owing to the greater presence of long-term hematopoietic stem cells on the fourth day.^19^ In a prospective, single-center cohort study, basal CD34+ cells and PLT counts before G-CSF administration were identified as predictors of the likelihood a sufficient number of CD34+ cells would be obtained on day 4 in healthy donors.^20^ Different studies might yield slightly divergent conclusions, potentially related to the variables included and algorithms used. Although univariate regression analysis indicated that age, BMI, and some routine hematological indices before collection are significant factors predicting a single successful collection, this is not conducive to clinical practice. Following application of the XGBoost algorithm and multivariate regression analysis, only Mono%, age, and CD34+ cell% were identified as the most significant predictors.

Age has been demonstrated in numerous studies to be a predictor of successful single apheresis collections and is associated with the threshold levels of CD34+ cells.^7,21,22^ However, age was not included in the predictive formula of PBSC yield in another study; instead, the model incorporated the peripheral blood CD34+ count and total volume processed.^23^ This bias serves as a reminder that different variables can lead to varying conclusions across different models. Therefore, it is essential to perform validation to ensure their reliability and applicability. Many pre-apheresis peripheral blood CD34+ cell count-based algorithms have been established since the 1990s. The “target value-tailored” apheresis formula revealed a correlation between the pre-apheresis peripheral blood CD34+ cell count and the quantity of CD34+ cells harvested, further precisely calculating the volume of blood to process.^24^ Based on this formula, a modified model exhibited an effective predictive ability in the validation cohort.^25^ Additionally, Karow and colleagues used regression analysis and developed an algorithm that included the pre-apheresis count of circulating CD34+ cells, the PBV, and the patient’s b.w. to predict the yield of CD34+ cells in pediatric patients.^26^ Mohty et al.^27^ presented a position statement suggesting that for autologous hematopoietic patients, having more than 20 CD34+ cells/μL in the peripheral blood before apheresis can serve as a trigger indicator, signifying sufficient CD34+ cells in the final collected product. However, the relative count of CD34+ cells depends on the results of flow cytometry, which requires time and is not conducive to clinical practice owing to delays in obtaining results. Interestingly, the importance score of Mono% appeared to surpass that of CD34+ cells% under the XGBoost algorithm in our study. Previous research has substantiated the critical role of monocytes in prediction. Yang et al.^28^ revealed that the monocyte count after mobilization was a reliable predictor of the PBSC yield among patients with hematologic malignancies. A higher monocyte count at the start of leukapheresis was associated with better initial collection outcomes, as validated in ROC curve analysis. Another study also showed that the pre-collection peripheral monocyte count is a significant predictor of successful PBSC harvest in patients with lymphoma after chemo-mobilization.^29^ However, no monocyte-based formula has been constructed to predict the quantity of harvested CD34+ cells. Considering the greater accessibility of monocyte parameters from routine blood analyses compared with CD34+ cells, we suggest that future research could explore potential use of the monocyte count or Mono% as alternative predictors in apheresis algorithms. This approach, however, would necessitate empirical validation through extensive sampling and multicenter studies to confirm its efficacy.

### Study limitations

In this study, we used machine learning methods and identified Mono%, age, and CD34+ cell% as significant factors predictive of successful PBSC collection on the first attempt, offering important insights for guiding the clinical collection of PBSCs. However, there are some limitations to our study. First, this study followed a retrospective design, which might cause limitations in data collection and potential bias issues. Second, although internal validation was conducted in this study and the ROC curve demonstrated effective predictive capabilities, further external validation in additional centers is required to confirm our conclusions. Third, owing to a lack of data on day 4 after G-CSF administration, we had a limited ability to determine the adequacy of 4 days of G-CSF administration. Fourth, our study did not provide specific cutoff values for Mono%, age, and CD34+ cell% to determine whether to initiate apheresis. These will be investigated in future research involving a larger sample size.

## Conclusion

In this study, we effectively applied machine learning methods to identify significant predictive factors for the first-attempt success in PBSC harvests. Key factors such as the percentage of pre-collection Mono%, donor age, and CD34+ cell% significantly influenced the likelihood of a successful initial harvest. These findings provide valuable insights that can enhance the efficiency of PBSC collection procedures by potentially reducing the number of apheresis sessions needed, thereby alleviating donor discomfort and lowering associated costs. However, the study’s retrospective design and absence of external validation highlight the need for further research. Additionally, the lack of data from day 4 of G-CSF administration and specific cutoff values for the identified predictive factors indicate areas for future study, which should involve more extensive datasets from multiple centers to establish robust predictive models. Future research could ultimately refine the guidelines for initiating apheresis and improve outcomes in clinical settings.

## Data Availability

The data are available from the corresponding author upon reasonable request.
